# Biodegradable bioadhesive nanoparticle incorporation of broad‐spectrum organic sunscreen agents

**DOI:** 10.1002/btm2.10092

**Published:** 2018-07-06

**Authors:** Hee‐Won Suh, Julia Lewis, Linda Fong, Julie Ying Ramseier, Kacie Carlson, Zheng‐Hong Peng, Emily Sara Yin, W. Mark Saltzman, Michael Girardi

**Affiliations:** ^1^ Dept. of Biomedical Engineering, Yale School of Engineering & Applied Science 55 Prospect Street, New Haven CT 06520; ^2^ Dept. of Dermatology, Yale School of Medicine 333 Cedar Street, New Haven CT 06520

**Keywords:** clinical study, drug delivery, nanoparticle, photodegradation, reactive oxygen species, sunscreen, ultraviolet radiation

## Abstract

Conventional emulsion‐based sunscreen formulations are limited by postapplication epicutaneous penetration that increases the risk of allergic dermatitis, cellular damage, and filter photodegradation upon ultraviolet radiation (UVR) exposure. Encapsulation of the UVB filter padimate O within bioadhesive biodegradable nanoparticles (BNPs) composed of poly(d,l‐lactic acid)‐hyperbranched polyglycerol was previously shown to enhance UVR protection while preventing skin absorption. Herein, we assess the capacity of BNP co‐incorporation of avobenzone and octocrylene to provide broad‐spectrum UVR protection. The ratio of UV filters within nanoparticles (NPs) was optimized for filter–filter stabilization upon UV irradiation and maximum drug loading. In vitro water‐resistance test showed significant particle retention at 85% over 3 hr. In a pilot clinical study, protection against UVR‐induced erythema of BNPs was found to be comparable to the FDA standard P2. Thus, sunscreen formulations utilizing BNP incorporation of a combination of organic filters may offer key safety and performance advantages.

## 1 INTRODUCTION

Ultraviolet radiation (UVR) is the major environmental risk factor for the development of cutaneous malignancies.^1^ UVR exposure can lead to acute skin damage, such as phototoxic erythema (i.e., sunburn), with a history of blistering sunburns associated with increased risk of malignant melanoma. Chronic UVR exposure is most notably associated with an increased risk of nonmelanoma skin cancer. In the United States, skin cancer is the most prevalent form of cancer, affecting one in five individuals with an annual economic burden estimated at $8.1 billion,^2^, ^3^, ^4^ making UVR exposure a major public health concern. Based on the relative contributions to types of skin damage, the solar UVR spectrum is divided into the major wavelength bands of UVA (400–320 nm), UVB (320–280 nm), and UVC (<280 nm). While UVB is the principal driver of phototoxic erythema and direct damage to genomic DNA (gDNA) via induction of cyclobutane pyrimidine dimers (CPDs),^5^ whereas UVA is the major cause of reactive oxygen species (ROS) generation including after absorption, excitation, and energy transfer by endogenous or exogenous chromophores that may indirectly also damage gDNA. Thus, effective UVR‐induced skin cancer prevention strategies must target both UVA and UVB.

There are two major types of sunscreens approved by the US Food and Drug Administration (FDA) for topical application: inorganic/physical (TiO_2_ or ZnO) particles and small organic molecules. UV protection and penetrance by inorganic particles can vary significantly with particle size.^6^ While the less conspicuous nano‐sized particles are more desirable for user compliance, light scattering ability decreases exponentially with size. Also, inorganic NPs can act as photocatalysts and generate ROS.^7^ Organic UV filters, largely substituted aromatic compounds, are commonly available as emulsions with surfactants and stabilizers. Due to the small size and lipid solubility of organic filters, their efficiency is limited by epicutaneous penetration and loss of effective concentration at the skin's surface.^8^ In addition, UV‐induced sunscreen degradation is associated with an increased risk of both irritant and allergic contact dermatitis,^9^ and spontaneous generation of ROS within and around compromised skin cells.^10^, ^11^ However, organic formulations are generally more esthetically appealing due to their inconspicuous appearance. Organic sunscreens absorb at various wavelengths, and commercial products often use a number of UV filters in combination to maximize broad‐spectrum protection. Organic UV filters comprise significant percentage of the total formulation by weight, necessitating solutions to overcome the drawbacks of conventional formulations.

To this end, numerous solutions have been explored. For example, UV filters can be conjugated directly onto polymers to form new UV absorbent material, such as polycrylene, Parsol SLX, and E‐Sal.^12^, ^13^, ^14^ However, these novel actives are not currently approved for use by the FDA. In fact, no new UV filters have been approved since 1997.^15^ For methods that use actives without covalent modification, solid NPs composed of various natural or synthetic materials can encapsulate the organic UV filters to slow down skin penetration of organic filters. NPs composed of silica,^13^, ^16^, ^17^, ^18^ lipids,^19^, ^20^, ^21^, ^22^, ^23^ gelatin,^24^ and synthetic polymers^25^, ^26^, ^27^, ^28^, ^29^, ^30^, ^31^, ^32^, ^33^ encapsulating both organic and inorganic UV filters,^34^, ^35^, ^36^, ^37^, ^38^, ^39^, ^40^ have been reported, but only characterized in vitro. Previously, we explored the advantages of encapsulating an FDA approved organic UVB filter, padimate O (PO), into biodegradable polymeric NPs composed of amphiphilic co‐block polymer, poly(d,l‐lactic acid)‐hyperbranched polyglycerol (PLA‐HPG). We demonstrated that this delivery platform forms nonadhesive NPs (NNPs) via self‐assembly, in which hydrophobic PLA core efficiently encapsulates small molecules while HPG coverage on the surface resists adhesion to proteins, thereby prolonging blood circulation^41^ and wide distribution when injected in solid tumors^42^ in other applications. To enhance topical retention of sunscreen formulation, NNPs were converted into bioadhesive NPs (BNPs) by selectively oxidizing terminal groups on HPG to aldehydes, resulting in significant retention of BNPs occurring at the skin surface via reversible^43^ Schiff base formation. Extracellular proteins rich in lysine residues^44^ provide amines necessary for covalent bonding interaction with the aldehyde end‐groups. Schiff base formation is implicated in various biochemical interactions, such as collagen cross‐linking^45^ and skin sensitization to small molecules.^46^ In our previous study, we applied fluorescent NPs topically to various substrates, including poly‐l‐lysine (PLL) coated slides, ex vivo pig skin, and in vivo mouse skin.^30^ In sharp distinction to NNPs, BNPs remained on the stratum corneum and resisted epidermal penetration. Furthermore, BNPs encapsulating PO reduced formation of free ROS, signs of ROS‐mediated toxicity, and induction of physiological changes (e.g., thickened orthokeratosis and epidermal hypertrophy) compared to control sunscreen upon UV irradiation. Our studies suggested that PLA‐HPG is a safe and versatile platform for delivery of small organic molecules.^41^, ^43^, ^47^

To extend these findings, we herein explored encapsulation of combinations of sunscreen actives in BNPs for clinical use by co‐encapsulating organic UV filters approved in the United States for broad‐spectrum protection. In vitro characterization of sunscreen‐NPs shows that UV filters can be co‐encapsulated with high encapsulation efficiency and loading, more than three‐fold higher than previously reported, with preferential encapsulation of the more hydrophobic UV filter. While UV filters are prone to degradation upon UV absorption, BNPs showed significant photostabilization of filters upon irradiation with a solar simulator. We also observed a significant decrease in free ROS with filter encapsulation. Water‐resistance test showed significant UV absorbance retention in the absence of additional formulation. Furthermore, we evaluated the BNPs for erythema prevention on human subjects against broad‐spectrum UVR, and showed that protection against phototoxic erythema using avobenzone (AVO)/octocrylene (OCR)‐BNP in water is at a level comparable to an FDA sunscreen standard in an optimized formulation.

## 2 MATERIALS AND METHODS

### 2.1 Materials

PLA (Mn = 11–12 kDa) was obtained from Lactel Absorbable Polymers. 1,1,1‐tris(hydroxymethyl)propane, potassium methoxide, 4‐dimethylaminopyridine (DMAP), N,Nʹ‐diisopropylcarbodiimide (DIC), dimethylformamide (DMF), diethyl ether (ether), and PO, and poly(l‐lysine) (PLL) were obtained from Sigma‐Aldrich. Glycidol, octinoxate, ethyl acetate (EtOAc), dimethyl sulfoxide (DMSO), dichloromethane (DCM), acetonitrile (ACN), acetone, methanol, deionized water, trifluoroacetic acid (TFA), phosphate‐buffered saline (PBS), DiIC_18_(5) solid (DiD), and dihydrorhodamine 123 (DHR123) were purchased from Thermo Fisher Scientific. Eusolex‐9020 and ‐OCR were obtained from EMD Millipore. Sunscreen purity was confirmed by gas chromatography mass spectrometry and high‐performance liquid chromatography (HPLC) analysis against secondary standards from Aldrich. VITRO‐SKIN^®^ was obtained from IMS, Inc., and HelioPlates HD6 polymethylmethacrylate (PMMA) plates from Labsphere. Aldehyde quantification assay (ab138882) was purchased from Abcam. Polymers HPG and PLA‐HPG were prepared using literature methods.^41^

### 2.2 Methods

#### 2.2.1 Particle preparation

UV filter‐loaded NNPs were synthesized via a modified single emulsion method.^41^ Briefly, 100.0 mg of PLA‐HPG was dissolved in 2.4 ml EtOAc overnight. UV filter (AVO, OCR, octinoxate, or combinations thereof, vide infra) or 0.2wt% fluorescent dye was dissolved in 0.6 ml DMSO and was added to the polymer solution, and the combined organic phase was added drop‐wise to 4 ml of vortexing water. The mixture was further emulsified using a probe‐sonicator for four‐cycles at 10 s interval at 0°C. The emulsion was immediately diluted in 10 ml of water with stirring, and EtOAc was removed via rotary evaporation at RT. NPs were collected via centrifugation at 4,000 *g* for 30 min at 4°C using a 100 kDa MWCO centrifugal filter, and washed twice with 15 ml water to isolate NNPs in ∼75% yield. NNPs were resuspended in 1 ml water and stored at −20°C.

Oxidative cleavage of terminal vicinal diols was achieved with modification of a previously reported procedure.^30^ NNP stock was diluted three‐fold with water to approximately 25 mg/ml concentration. One volume of NNPs in water was incubated with 1 vol. of 0.1 M NaIO_4_(aq) and 1 vol. of 10× PBS on ice for 20 min. The reaction was quenched with 1 vol of 0.2 M Na_2_SO_3_(aq) and the resulting BNPs were isolated using a 100 kDa MWCO centrifugal filter. NPs were washed twice with 15 ml of water, resuspended in water, and stored at 4°C.

#### 2.2.2 Particle characterization

The hydrodynamic size by dynamic light scattering (DLS) and zeta potential (ZP) measurements of NPs in water were acquired via Zetasizer Nano‐ZS (Malvern) at RT. The stability of NPs was monitored at 4°C for 2 months also by DLS. Transmission electron microscopy (TEM) images were collected on a Tecnai Osiris electron microscope (FEI) with an accelerating voltage of 120.0 kV. NP samples were prepared on 400‐mesh‐type Cu grids with carbon coating (Electron Microscopy Sciences) with 0.2% uranyl acetate staining, and dried under ambient conditions before imaging.

Fluorescence of DiD‐NPs was monitored using SpectraMax M5 plate reader (Molecular Devices, Ex/Em: 644/665 nm). The amount of sunscreens encapsulated in NPs was determined by RP‐HPLC (Shimadzu) with Microsorb‐MV C18 column (250 × 4.6 mm, 100 Å) (Agilent) at 30°C. The analysis was performed using the mobile phase 0.1% TFA in ACN and 0.1% TFA in water. Absorbance was monitored at 313 nm. A typical sample was prepared by dissolving a known amount of NPs in ACN and filtering through a 0.45 μm filter (Acrodisk). The loading and encapsulation efficiency was calculated against a standard curve.

$$UV\;filter\;loading\;\left(\%\right)=\;\frac{mg\;of\;UV\;filter\;encapsulated}{mg\;of\;NP}\;\times \;100\%$$

$$Encapsulation\;efficiency\;\left(\%ee\right)=\frac{mg\;of\;UV\;filter\;encapsulated\;in\;NPs}{mg\;of\;sunscreen\;initially\;added}$$

In vitro drug release was analyzed by incubating the NPs in 0.5 ml 0.25% Tween 20 (aq) at RT in a 100 kDa MWCO centrifuge filter tube. At each time point, the particles were filtered at 3,500 g for 10 min, the NPs were resuspended in 0.5 ml of fresh Tween solution, and the filtrate was analyzed via HPLC.

#### 2.2.3 Photostability of nanoparticles in vitro

Photodegradation of AVO and/or OCR loaded particles was tested using a solar simulator (Daavlin 4 panel), with UVA (320–400 nm) output of 62.6 W/m^2^ and UVB (280–320 nm) of 3.6 W/m^2^, measured at a working distance of 9 in. from the light source. A concentration of 0.2 mg/mL NPs in water was used to avoid distortion in the spectrum due to saturation. Background absorbance of deionized (DI) water was subtracted from the NP absorbance. The plate was sealed and irradiated over the panel. At each time point, UV absorbance was monitored at 304 nm (OCR) and 360 nm (AVO), and as a spectrum between 280 and 400 nm, on a UV spectrophotometer using a Greiner UV‐Star^®^ microplate.

In addition, UV absorbance of NPs was measured on a dry surface using PMMA plates with rough, skin‐like, topography. NPs were added drop‐wise across the 5 × 5 cm PMMA plate and spread evenly with a gloved finger. Samples were left to dry for 15 min before measurement using Labsphere UV‐2000S UV transmittance analyzer at four different locations. Photodegradation after 2 hr at 800 J/m^2^ of UVB light was compared to the initial measurement, and the retention of total absorbance was calculated.

$$Total\;Abs=\sum_{x\;=\;291nm}^{x\;=\;399\;nm}Abs(x)+0.5[Abs\left(290\right)+Abs\left(400\right)]$$

$$\%\;retention=\left(1-\frac{Total\;Abs\left(post-treatment\right)}{Total\;Abs\;\left(pre-treatment\right)}\right)\times 100\%$$

#### 2.2.4 Effect of nanoparticle encapsulation on ROS generation

Sunscreen‐NPs (1:3‐AVO/OCR‐NPs) and 1:3‐AVO/OCR emulsion in water were incubated in the dark in a UV‐transparent 96‐well plate with ROS scavenger DHR123. After 20 min, the plate was exposed to UV light from the Daavlin panel as previously described. Fluorescence intensity of rhodamine 123 (R123) in each well was monitored using a plate reader up to 1 hr, and was baseline‐corrected by fluorescence from oxidation of DHR123 alone at each time point.

#### 2.2.5 Water resistance of nanoparticles

VITRO‐SKIN^®^ was hydrated following manufacturer's protocols, treated with PLL, then mounted on a glass slide and 10 mg/ml NPs in water (DiD‐NNPs, DiD‐BNPs, or AVO/OCR‐BNP) applied dot‐wise at 2 mg/cm^2^, spread evenly with a gloved finger, and left to dry for 15 min before analysis.

For fluorescent samples, images at four random fields in grid per sample were acquired on a Leica SP5 confocal microscope. For sunscreen samples, UV absorbance of AVO/OCR‐BNPs on VITRO‐SKIN^®^ was measured at four distinct locations using the UV transmittance analyzer before securing the sample inside a 4 L water bath with stirring at 150 rpm for 3 hrs. Final absorbance was measured to determine absorbance retention.

#### 2.2.6 Determination of sun protection factor

The following procedures were performed with informed consent from all volunteers, in accordance with a protocol approved by the Yale Human Investigation Committee. This study was an interventional, nonrandomized trial, with focus on UV‐induced erythema with or without protection to characterize the sun protection factor (SPF) of BNPs in healthy volunteers with fair skin of Fitzpatrick skin type I (always burn easily, never tans) and II (always burn easily, tans minimally).

The UV source was a Model 601 Multiport^®^ SPF Testing Solar Simulator from the Solar Light Company, Inc., which meets the latest FDA spectral irradiance standards for broad‐spectrum light source (290–400 nm). The multiport light source consists of six adjustable channels (liquid light guides), which were set to 1.15^n^ dose increments (each dose 15% greater than the previous), and calibrated before and after each exposure. A hypoallergenic foam applicator (5.3 × 4.2 cm) was placed directly on the test site and multiport light guides were inserted into the applicator until the light source touched the subject's skin. FDA approved active comparator P2, which contains 7% PO and 3% oxybenzone in a cream formulation, was purchased from Cosmetech Laboratories, Inc. and used as a control. The expected SPF for the standard is 16.3 (accepted range: 13.7–17.7).

The protocol was extended over three visits. During the first visit, we assessed the inherent minimal erythema dose (MED) on unprotected skin (MEDu) for each subject by administering UVR at six doses in increasing geometric series of 1.15^n^. MEDu dose was determined 24 hr postirradiation as the UV dose that gave the first perceptible erythema with delineated borders. Subsequently, 2 mg/cm^2^ each of AVO/OCR‐BNPs or P2 standard was applied to two clearly defined sections on subject's back, and spread evenly using a finger cot. Both finger cots and weighing receptacles were weighted before and after use to ensure correct recording of dosage. Application area was let to dry for 15 min, and irradiated based on the predetermined MEDu and expected SPF. A second MEDu test was conducted concurrently to confirm the MEDu. UVR was administered to each of the three delineated sites on subject's back. MED of protected skin (MEDp) and second MEDu were assessed 24 hr after UVR exposure. SPF was determined for each test subject as the ratio of MED for the protected subsite to the MED for the unprotected subsite (MEDu/MEDp). Skin reaction at the site of application was assessed by physical exam, and monitored over 1 week.

## 3 RESULTS

### 3.1 Development of broad‐spectrum sunscreen nanoparticles

**Figure 1:**
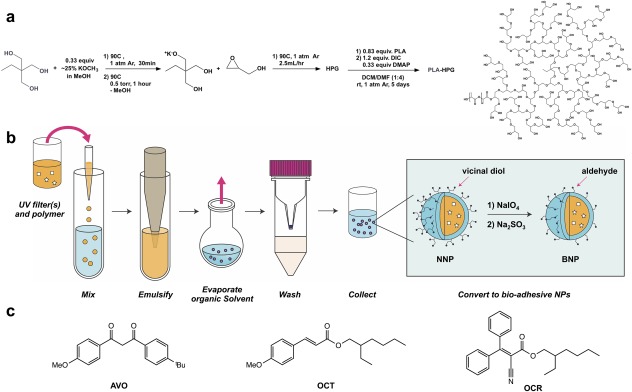
(a) Synthetic scheme of PLA‐HPG. (b) Synthetic scheme of PLA‐HPG nanoparticles and surface modification to BNPs. (c) Molecular structure of avobenzone (AVO), octinoxate (OCT), and octocrylene (OCR)

Amphiliphilic co‐block polymers, such as PLA‐HPG, make efficient vehicles for delivery of hydrophobic small molecules, such as organic UV filters. An additional advantage of HPG is the availability of vicinal diols, which can be converted to bioadhesive aldehydes. Previously, we demonstrated that PLA‐HPG can encapsulate 10wt% of FDA approved organic UV filter PO with high encapsulation efficiency. PO has favorable physical properties, such as high hydrophobicity and extinction coefficient, complemented by safer delivery with BNPs; however, PO‐BNPs exhibit poor absorption in the UVA region. In addition, PO is rarely used in commercial sunscreen products due to its association with PABA‐derivative induced adverse reactions, such as contact dermatitis.^48^ Since UVA radiation contributes significantly to carcinogenesis as well as skin aging,^49^ we explored encapsulation of both UVA and UVB filters to further evaluate the scope of our NP platform. HPG and PLA‐HPG copolymer was synthesized (Figure 1a) and particles were formulated via a single emulsion‐solvent evaporation method (Figure 1b) as previously described.^30^ UV absorbance of UV filter‐loaded NPs shows that PLA‐HPG can be used to efficiently encapsulate not only PO, but also other common UV filters (Supporting information Figure S1). UV filter encapsulation was determined by calculating the weight percentage of UV filters in lyophilized solid NPs as determined by HPLC (Supporting information Table S1). Our results show that other organic UV filters have comparable loading and encapsulation efficiencies to PO, and the resulting NPs do not show any significant solvatochromic shift in absorbance.

### 3.2 Temporal changes in absorption spectrum upon irradiation

UV filters are prone to UV‐induced isomerization, and subsequent decomposition, which can affect the absorption spectrum of a sunscreen over the duration of sun exposure.^10^ In particular, photodegradation of AVO, the most widely utilized FDA approved UVA filter, has been well documented.^50^ AVO degradation has been attributed to excitation from the triplet state of its keto form to generate numerous photoproducts.^51^, ^52^, ^53^

**Figure 2:**
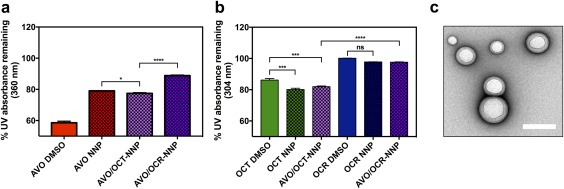
(a) UV absorbance retention of AVO‐, OCT‐, OCR‐, AVO/OCT‐, and AVO/OCR‐NPs after 2 hr of UV irradiation analyzed at λmax of AVO (360 nm). (b) UV absorbance retention of UVB filter post‐UV irradiation analyzed at λmax of respective UVB filters (OCT in NP: 306 nm, OCT in oil: 290 nm, OCR in NP: 304 nm). (c) Representative TEM of 1:1‐AVO/OCT‐NP (Scale bar: 200 nm)

Triplet state quenchers, including antioxidants^54^ and other UV filters,^55^ can interact with AVO and enhance its photostability. Therefore, we tested the photoreaction of combinations of UV filters by co‐encapsulating AVO with OCT or OCR in 1:1 ratio, and comparing the performance with that of single‐drug‐encapsulating NPs. UV filter‐encapsulated NPs (10% AVO, OCT, OCR, or 5% each of AVO/OCT or AVO/OCR) in water were irradiated with a solar simulator for 2 hr, and the decrease in absorbance at λ_max_ for each sample was determined using UV‐Vis spectroscopy (Figure 2). Notably, we observed that NP encapsulation increased the photostability of AVO over AVO in DMSO (polar aprotic solvent) or mineral oil (nonpolar solvent) solutions (Supporting information Figure S2). This is similar to stabilization of OCT in poly(d,l‐lactic‐co‐glycolic acid) and ethyl cellulose NPs reported by Perugini and colleagues.^26^ In addition, we observed significant photoprotection of AVO in AVO/OCR‐NPs compared to AVO/OCT‐NPs. This observation is consistent with that of Lhiaubet‐Vallet and colleagues, who observed similar cooperative or destructive filter–filter interaction in miglyol upon irradiation.^55^ The instability of OCT is attributed to a rapid isomerization process into Z‐isomer, which is susceptible to further irreversible formation of cycloaddition products or degradation.^56^

**Figure 3:**
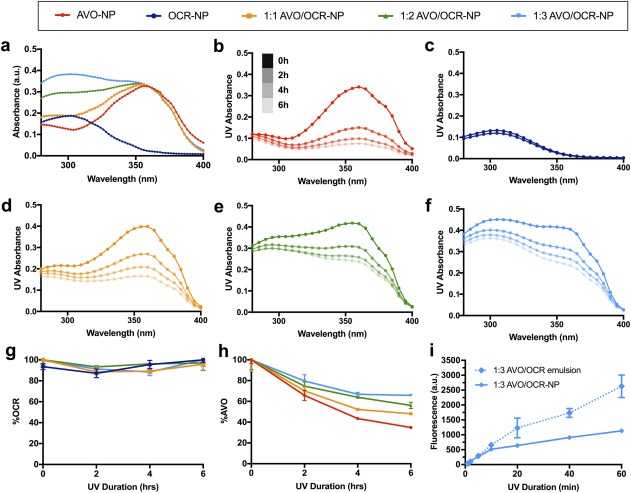
Photoprotection of OCR on AVO in NPs. (a) Initial absorption spectra of NPs. (b‐f) Temporal change in the UV absorbance of NPs under UV irradiation of (b) AVO, (c) OCR, (d) 1:1 AVO/OCR, (e) 1:2 AVO/OCR, (f) 1:3 AVO/OCR. (g) Percent remaining of OCR by HPLC. (h) Percent remaining of AVO by HPLC. (i) ROS was measured by fluorescence of R123 in UV irradiated samples of 1:3‐AVO/OCR‐NP or emulsion at each time point

A combination of two or more sunscreens in varying ratios is commonly used to increase the level of photoprotection (each drug within the maximum FDA limit) and achieve neutral density absorption across the UV spectrum. An ideal sunscreen would maintain a consistent absorption profile over time, and therefore provide continuous coverage over action spectra within the skin.^57^ To this end, we co‐encapsulated three different ratios of AVO to OCR in NPs to study the effect of OCR concentration on absorption profile and filter–filter interaction (Figure 3). Sunscreen‐NPs with approximately 10% total of AVO/OCR in various ratios (0:1, 1:0, 1:1, 1:2, or 1:3) were prepared and irradiated with a solar simulator. UV absorbance of each sample was monitored at 2, 4, and 6‐hr time points (Figure 3b‐f and Supporting information Table S2). All AVO‐containing NPs exhibited significant loss of absorption at 360 nm, while OCR‐NPs showed more robust and persistent absorption spectra. HPLC analysis of aliquots from the UV irradiated samples (Figure 3g,h) confirmed the photostabilizing effect of OCR on AVO in our NP formulation, with twice the concentration of AVO detected in 1:3‐AVO/OCR‐NPs compared to AVO‐NPs at the end of the experiment. Light scattering by the NPs can affect sunscreen‐NP absorbance; however, we found that the background absorbance of NPs without any drug encapsulated is not significantly different from that of water (control) at the concentration used in this experiment (Supporting information Figure S3). Of note, 1:3‐AVO/OCR‐NPs also showed the most consistent broad‐spectrum UV absorption profile between 290 and 400 nm over the 6‐hr period (Figure 3f).

UV‐induced chemical excitation of cosmetic formulations and UV filters has been implicated in the generation of ROS and free radicals, facilitating phototoxic and photoallergic effects.^11^ In addition, excited aromatic hydrocarbons are prone to triplet state quenching by molecular oxygen, leading to ROS induction.^58^ In our previous work, we determined that NP encapsulation of PO reduced free ROS detected after UV exposure.^30^ Also, we observed that NP encapsulation slowed UV filter degradation, possibly by creating an environment for poor stability in excited state. In addition, reduction of ROS upon UV irradiation by physical entrapment of UV filters has been demonstrated in ZnO/CeO_x_ microspheres.^39^ Therefore, we hypothesized that our AVO/OCR‐NPs may also exhibit a similar reduction in free ROS. Thus, we incubated the 1:3‐AVO/OCR‐NPs as well as UV filter‐only emulsion in water (with trace DMSO) with the ROS probe DHR123 and irradiated with a solar simulator (vide supra). Fluorescence of samples relative to fluorescence of DHR123 alone incubated in water was recorded at 2, 5, 10, 20, 40, and 60 min postirradiation (Figure 3i). NP encapsulation significantly reduced the amount of ROS generated compared to UV filter emulsion in water, suggesting that NP encapsulation either interferes with production or hinders release of ROS, possibly by retardation of filter degradation or ROS confinement.

### 3.3 Characterization of nanoparticles

**Figure 4:**
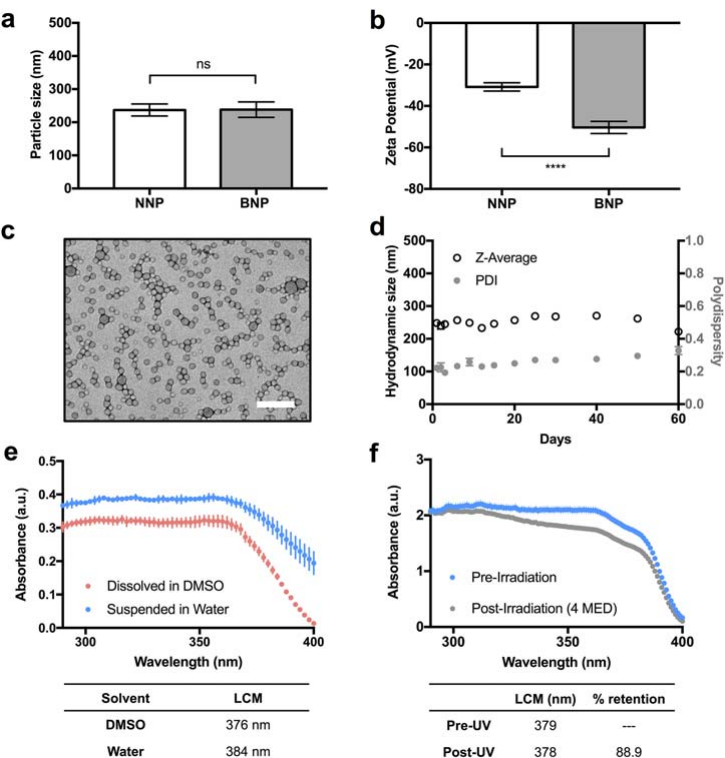
Characterization of nanoparticles used in clinical studies. (a) Hydrodynamic size distribution of 1:3‐AVO/OCR‐NNPs and BNPs at maximum loading (*n* = 9) (b) Comparison of ZP of NNPs and BNPs. Each dot represents an average three readings from one batch. (c) TEM image of 1:3‐AVO/OCR‐BNPs (Scale bar: 200 nm). (d) BNP stability at 4°C over 2 months. (e) UV absorbance of 0.01% BNPs in DI water vs. DMSO (dissolved). (f) UV absorbance retention of 35% NPs measured on PMMA plate before and after 800 J/m^2^

In a given sunscreen formulation, the maximum percentage of sunscreen (by weight) approved by the FDA varies from compound to compound. For PO, it is 10% (100 mg PO/1g formulation); AVO, 3%; and OCR, 10%. This is orders of magnitude more concentrated than the previously reported PO concentration in BNPs, which protected against UV‐induced CPDs at 1 mg/mL NP concentration (0.01% PO) in murine skin. In order to challenge the scope of our PLA‐HPG NPs, we tested for maximum 1:3 co‐encapsulation of AVO/OCR (Supporting information Table S3). Maximum 1:3 loading of AVO/OCR was determined as 29wt% UV filter (initial feed of 10 mg AVO and 30 mg OCR in 100 mg PLA‐HPG). NNP to BNP conversion was performed as previously described, by treatment of NNPs with NaIO_4_.^30^ NP size does not change appreciably from NNP to BNP conversion (Figure 4a), whereas surface‐potential became significantly more negative (Figure 4b). NP characterization is further summarized in Table 1. Aldehyde conversion of vicinal diols was monitored with a fluorometric aldehyde quantification assay, and saturation was observed past 15 min (Supporting information Figure S4), similar to that observed previously.^30^, ^43^ TEM imaging of NPs shows consistent spherical morphology even at ∼30% encapsulation of actives (Figure 4c). TEM imaging suggested a small degree of aggregation; however, when the stability of the NPs was monitored in solution by DLS, the BNPs remained a stable suspension in water with low polydispersity (PDI) for over 2 months in the absence of additives, even at 35 wt% NP concentration (wt/vol) or approximately 3% AVO and 9% OCR concentrations (Figure 4d). Given these observations, the aggregation suggested by TEM is most likely an artifact produced by drying during sample preparation. BNP size (Z‐avg) and PDI showed a small positive correlation (*r* = .72, *p* < .01 and *r* = .89, *p* < .01, respectively). Drug release from BNPs in DI water was below the limit of detection (by HPLC) over 6 months. Minimal release was observed even when the particles were resuspended in aqueous solution of Tween 20, a surfactant, which generally facilitates increase in the rate of release (Supporting information Figure S5).

**Table 1:** Characterization of nanoparticles used in clinical study

NP	Z‐Avg (nm)	Pdl	Zeta potential (mV)
NNP	222	0.198	−29.2
BNP	216	0.169	−53.3
**Drug**	**% loading (Exp)**	**% loading (Theor)**	**%EE**
AVO	7.8 ± 0.58	7.1	>99
OCR	22.7 ± 1.7	21.4	>99

We further probed the effect of solvent on 1:3‐AVO/OCR‐NP absorbance by preparing samples of equal quantities that were either suspended in water or dissolved in DMSO. We observed that UV filters are less efficient absorbers in DMSO than in NPs in water (Figure 4e), consistent with our previous findings.^30^ This could also suggest a significant effect of solvent on sunscreen absorbance,^59^ that can obfuscate analysis of in vitro characterization of NP formulations for in vivo efficacy.

In order to compare the absorbance profile in water suspension versus on application, we acquired the in vitro UV transmittance of this sample on a polymer substrate (PMMA) using the Labsphere UV transmittance analyzer (Figure 4f). UV absorption spectrum was found as an average of four locations, reflecting uniformity of application. BNPs in water were evenly applied at 2 mg/cm^2^, consistent with the FDA sunscreen monograph.^60^ Neutral density protection was observed between 280 and 380 nm with 90% of the UV absorbance (area under the curve) occurring below 379 nm, a wavelength characterized as the lambda critical mean (LCM). A minimum value of 370 nm of LCM is required to categorize a formulation as a broad‐spectrum sunscreen, with higher value associated with better UVA protection profile. Our NP fits this description of a broad‐spectrum sunscreen. Subsequently, PMMA plates were subjected to 800 J/m^2^ of broad‐spectrum UVR. A slight downward slope emerged between 310 and 360 nm, as a result of AVO degradation. However, the LCM remained 378 nm, with 89% of area under the curve preserved postirradiation. This retention of spectral absorption profile of NPs on surface is consistent with that in water suspension (Figure 3f).

### 3.4 In vitro water‐resistance tests

There are three major international standards for evaluation of water resistance in vivo: Europe (COLIPA), Australia (AS/NZS), and United States (FDA).^61^ There is no standardized in vitro alternative for water‐resistance testing,^62^ but the use of nonbiological substrates are more desirable.^63^ PMMA plates have been reported to give reliable results for photostability testing, but not for water‐resistance testing compared to parallel in vivo results.^64^, ^65^, ^66^, ^67^ Herein, we used an alternative skin‐like polymeric substrate, VITRO‐SKIN^®^, a synthetic skin substrate with similar properties (topography, surface tension, ionic strength, pH) to human skin following manufacturer's protocol for long lasting water resistance. Studies using VITRO‐SKIN^®^ substrates have shown that a laminar flow of 150 rpm up to 60 min can reliably predict the in vivo testing conditions within reasonable limits.^68^

**Figure 5:**
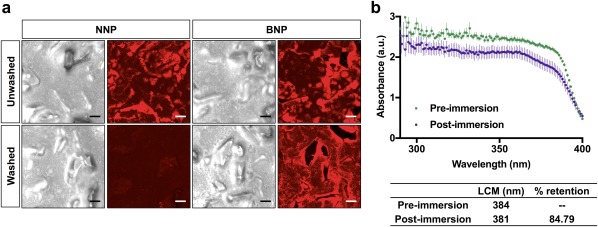
Water resistance of sunscreen nanoparticles. (a) Retention of NNPs and BNPs on PLL‐coated VITRO‐SKIN^®^. (b) UV absorbance retention after water‐resistance test at 150 rpm for 3 hr

First, we investigated the adhesion and retention of fluorescent NPs on VITRO‐SKIN^®^. We coated the surface of VITRO‐SKIN^®^ with PLL to provide a higher amine density similar to the extracellular protein rich environment of the stratum corneum that allows robust covalent BNP‐skin interaction.^30^ NPs loading 0.2% hydrophobic dye (i.e., DiD‐NNPs and DiD‐BNPs) were prepared and characterized as previously reported, and 1% wt/vol NPs in water were applied on VITRO‐SKIN^®^ at 2 mg/cm^2^ application density. We used 2 mg/cm^2^ as our application density to ensure application of a comparable dose between SPF testing and water‐resistance testing. We tested the water resistance of surface bound particles by washing the NP‐treated substrate in a water bath for 10 s, and qualitatively comparing the NP fluorescence on washed and unwashed substrates using confocal microscopy. Representative images show uniform application of both NNPs and BNPs (Figure 5a, and Supporting information Figure S6). NNPs were easily removed with water, as they lack aldehyde groups that allow bonding with the substrate surface, and contain more hydroxyl groups that allow better suspension in water. In contrast, BNP‐treated substrate retained significant fluorescent NP coverage after washing.

We also tested the adhesion of UV‐active BNPs on PLL‐coated VITRO‐SKIN^®^. 1:3‐AVO/OCR‐BNPs at 35% wt/vol was applied at 2 mg/cm^2^ on prepared VITRO‐SKIN^®^, and left to dry. The substrate was held completely submerged in a water bath with stirring at 150 rpm at 30°C for 3 hr. Initial and final absorbance of the substrate was collected using the UV transmittance analyzer (Figure 5b). The average area under the curve was determined to quantify the degree of particle retention after water immersion. Our results indicate that even when BNP‐treated substrate was challenged to harsh washing conditions, ∼85% of UV absorbance was preserved with only a 3 nm decrease in LCM. The minimal LCM decrease suggests that the two UV filters are evenly encapsulated and, as a result, the neutral density absorption is not compromised upon water‐resistance testing across the UV spectrum, with no significant bias in loss of absorption at certain wavelengths.

### 3.5 Photoprotective effect of BNPs

**Figure 6:**
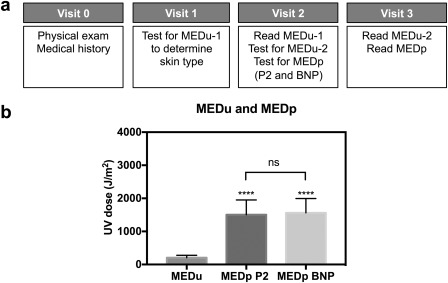
(a) Clinical study timeline. (b) UV dose to induce MED on unprotected (MEDu) versus protected (MEDp) skin. Sunscreen‐NP protection is comparable to high SPF (15) Standard P2

The performance of NPs co‐encapsulating AVO and OCR at the most favorable 1:3 ratio was tested in a pilot clinical study. The evaluation was performed in a controlled study with informed consent of 10 volunteers of both men and women of ages between 20 and 75 years of Fitzpatrick's skin types I and II. Each subject received 2 mg/cm^2^ of P2 sunscreen standard (7% PO and 3% oxybenzone)1 and BNPs in water (3% AVO and 9% OCR) on the lower back within respective delineated areas. Treated areas were exposed to UV irradiation from a solar simulator according to predetermined MED of unprotected skin of each subject (MEDu). The minimal erythema causing UV dose for P2 or BNP‐protected skin (MEDp) was recorded for each subject. The BNP‐based sunscreen performed at a level comparable to the P2 formulation (Figure 6). Our MEDu measurements were reproducible within error (Supporting information Figure S7). Dermal irritation was not explicitly tested in this study. However, no adverse skin reactions from photosensitivity or photoallergy were observed in subjects with application of BNPs by clinical examination in the duration of our studies. These preliminary results demonstrate that our AVO/OCR‐BNPs approximate the P2 standard of broad‐spectrum sunscreen and appear safe for topical application.

## 4 DISCUSSION

The study of novel drug delivery vehicles necessitates a comprehensive analysis of both in vitro and in vivo properties for safe application of the new technology. Here, we have demonstrated the scope of a PLA‐HPG‐based BNP platform for common UV filters approved in the United States. NPs with a hydrophobic PLA core and hydrophilic HPG corona were found to be highly compatible with hydrophobic organic small molecules, and screening of various UV filter combinations revealed that NPs can load up to 30% actives with limited release over time. We also found that two UV filters could be simultaneously encapsulated in BNPs to provide broad spectral coverage for enhanced UVA and UVB protection and filter stability. Consistent with our previous study, encapsulation of UV filters enhanced the reduction of free ROS, possibly through slower degradation of actives as well as active containment of ROS inside the particles.

Drug delivery with BNPs is an accessible, and scalable technology that may be used to improve both the function and esthetics of consumer products. One major advantage of BNP encapsulation of sunscreens is that it allows for flexible formulation of actives in a water‐based medium without addition of oil or organic solvent, compared to conventional oil‐in‐water (o/w) or water‐in‐oil‐in‐water (w/o/w) emulsion, which may greatly influence the esthetics of the formulation. Also, HPG coverage of the NP surface allows NPs to remain suspended in water without phase separation for an extended period of time. Furthermore, bioadhesive modification of the NP surfaces provides a truly topical delivery of sunscreen actives, which is a significant advantage as it may greatly reduce possible systemic absorption of UV filters.^30^ The surface modification of terminal HPG to aldehydes enables BNPs to covalently bind to the stratum corneum, thereby greatly reducing the potential absorption of UV filters into the living skin and systemic circulation.^30^ The sunscreen comparator P2 is an emulsion of active ingredients (PO and oxybenzone) with various inactive stabilizers, surfactants, and preservatives (e.g., sorbitol, lanolin, glyceryl stearate, stearic acid, and parabens). This type of formulation inherently facilitates the cutaneous absorption of actives. However, when encapsulated within our preformed NPs, the actives are immobilized within the polymer matrix providing long‐term stability and retaining high topical concentration upon application.

Water‐resistance testing showed that strong agitation of water is not sufficient to dislodge the BNPs in significant quantities. However, our previous studies have shown that NPs can be easily removed by rubbing with a towel.^30^ Future work can examine BNP bonding interactions by measuring the kinetics of Schiff base formation and hydrolysis from a lysine‐rich substrate under various conditions (e.g., sheer, pH). Understanding the conditions for detachment of NPs will inform performance under real‐life conditions in in vivo clinical studies for water‐resistance and long‐lasting protection. In addition, it will also be important to study the effect of water concentration in the formulation and the extent of NP bonding interaction with the skin using different delivery methods. Commercial sunscreen formulations include not only wet formulations such as creams and gels, but also dry solids such as sticks and powders. Therefore, it will be relevant to investigate the stability and activity of BNPs in a spectrum of aqueous to dry environments. While Schiff base synthesis for small molecules is often performed in dry organic solvents, we predict the bond formation will be significantly deterred in a solid formulation.

The BNP platform could be utilized to encapsulate other organic molecules, such as fragrances or antioxidants, or inorganic molecules, and enhance compatibility of UV filters with such molecules in the same product, in particular by using an alternative core material to PLA. Stability of the NPs may vary with loading of different active ingredients. In particular, some ingredients could influence the crystallinity of the core, and thus, mechanical studies of fluidity and deformation are of interest moving forward.

## 5 CONCLUSION

BNPs provide an attractive platform for delivery of a number of UV filter actives approved by the FDA, either alone or in combination, to human skin. The sunscreen‐NPs were evaluated in vitro for relevant performance characteristics, such as stability, photoprotection, and water resistance. In particular, co‐encapsulation of actives enhanced the broad‐spectrum protection of the NPs while delaying the photodegradation of actives. We obtained compelling evidence that UV filter‐loaded NPs alone in water can achieve protection against UVR that is comparable to an FDA approved sunscreen standard in the absence of additional formulation. In this pilot clinical study, BNPs were used as a suspension in water, which is not optimal for the even dispersion of NPs over time. Optimization of a more appropriate formulation may therefore further enhance their performance. Nonetheless, these results indicate that polymeric NPs as vehicles for organic UV filters may provide a biocompatible solution integral to safer and more efficient UV protection, and demonstrate a pathway to further improve current sunscreen formulations.

## Supporting information

Additional Supporting Information may be found online in the supporting information tab for this article.

Supporting Information 1
