# Supplementary material for: Biodegradable bioadhesive nanoparticle incorporation of broad‐spectrum organic sunscreen agents
Source: Bioeng Transl Med. 2018 Jul 6;4(1):129–40. doi: 10.1002/btm2.10092 (PMC6336670; doi:10.1002/btm2.10092)

Supplementary Information

Biodegradable Bioadhesive Nanoparticle Incorporation of Broad-Spectrum Organic Sunscreen Agents

Hee-Won Suh[1], Julia Lewis[2], Linda Fong[1], Julie Ying Ramseier[2], Kacie Carlson[2], Emily Sara Yin[2], W. Mark Saltzman[1*], Michael Girardi[2*]

[1] Department of Biomedical Engineering, Yale School of Engineering & Applied Science
55 Prospect Street, New Haven, CT 06511, USA
[2] Department of Dermatology, Yale School of Medicine
333 Cedar Street, New Haven, CT 06520, USA
*Contributed equally to this work

INDEX

**Figure S1**	Encapsulation of dye, AVO, OCT, and OCR in NPs
**Table S1**	Quantification of sunscreen loading in NPs
**Figure S2**	Degradation of AVO in DMSO and mineral oil
**Table S2**	AVO/OCR loading in NPs (HPLC)
**Figure S3**	Absorbance of blank PLA-HPG NPs in DI water
**Table S3**	Variation in Loading and %EE Dependent on the Ratio of AVO/OCR
**Figure S4**	Saturation of aldehydes quantified by fluorimetric aldehyde quantification assay
**Figure S5**	Release of sunscreens from 1:3 AVO/OCR co-encapsulated nanoparticles in 0.25% Tween
**Figure S6**	(a) Preparation of VITRO-SKIN®. (b) Fluorescence of PLL treated or untreated VITRO-SKIN® applied with NNPs or BNPs
**Figure S7**	Reproducibility of MED tests

Figure S1. Encapsulation of dye, AVO, OCT, and OCR in NPs

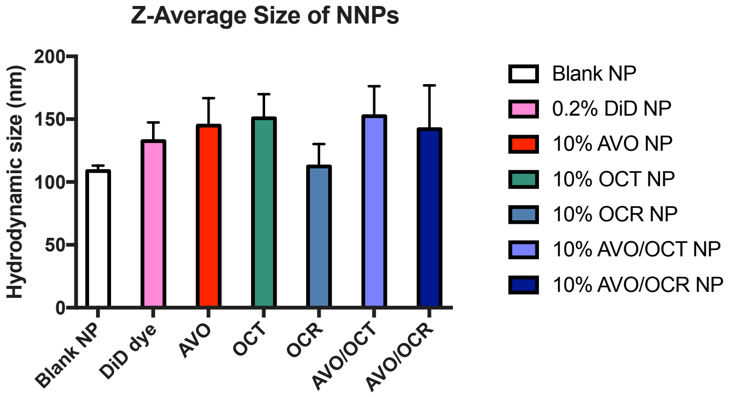

Table S1. Quantification of sunscreen loading in NPs

UV filter	Size (d. nm)	PdI	ZP (mV)	Loading (%)
**No filter**	108.1	0.186	-21.7	N/A
**DiD**	132.6	0.211	-30.4	0.2
**AVO**	144.9	0.248	-34.1	11.5 ± 1.3
**OCT**	150.7	0.272	-28.0	8.9 ± 0.9
**OCR**	112.5	0.243	-29.9	9.8 ± 1.4
**AVO/OCT**	152.3	0.221	-31.6	5.9 / 5.1 ± 0.7
**AVO/OCR**	142.1	0.261	-35.4	4.7 / 5.3 ± 0.7

Figure S2. Degradation of AVO in DMSO and mineral oil

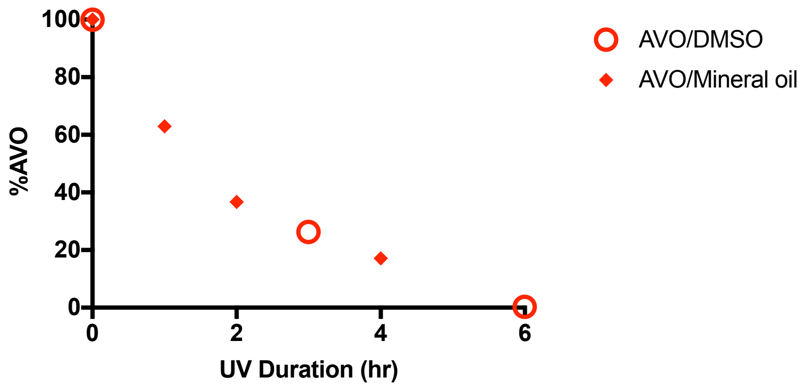

Table S2. Amount of UV filters (wt%) in NPs from **Figure 3a** determined by HPLC. Sunscreen-NPs with approximately 10% total of AVO/OCR in various ratios (0:1, 1:0, 1:1, 1:2, or 1:3) were prepared.

	**% Loading in NPs by HPLC**		**Filter ratio**
	**OCR**	**AVO**	**AVO/OCR**
**OCR**	8.41	0	1:0
**AVO**	0	9.80	0:1
**1:1**	4.00	4.04	1:1
**1:2**	6.44	3.13	1:2
**1:3**	6.61	2.24	1:3

Figure S3. Absorbance of blank PLA-HPG NPs in DI water at various concentrations (1.0 mg/mL – 0.1 mg/mL). Background absorbance of DI water was subtracted as in Figure 3a. Light scattering of blank NPs are small at low concentrations (< 0.01 a.u. at 0.2 mg NP/mL).

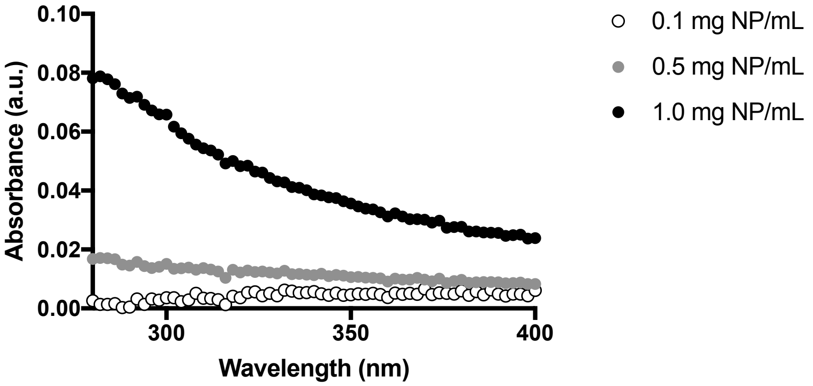

Table S3. Variation in Loading and %EE Dependent on the Ratio of AVO/OCR. The NP synthesis procedure relies on the self-assembly of NPs with co-precipitation of hydrophobic UV filters, AVO and OCR, encapsulated within the PLA core. As we increased the amount of AVO and OCR, we observed preferential encapsulation of OCR in NPs, skewing the encapsulation ratio

Starting ratio AVO/OCR/PLA-HPG (mg)	Drug	%loading	Ratio
5/10/100	AVO	3.2	1 : 1.98
	OCR	6.4	
10/20/100	AVO	7.4±0.28	1 : 2.24
	OCR	16.45±0.35	
15/30/100	AVO	15.05±0.07	1 : 2.05
	OCR	31.1±0.14	
20/40/100	AVO	10.5±0.28	1 : 2.27
	OCR	23.4±0.14	
10/30/100	AVO	6.4±0.42	1 : 3.1
	OCR	19.6±1.41	
12.5/37.5/100	AVO	6.6	1 : 3.36
	OCR	22.3	
15/45/100	AVO	7.9	1 : 3.4
	OCR	26.8	

Figure S4. Saturation of aldehydes quantified by fluorimetric aldehyde quantification assay

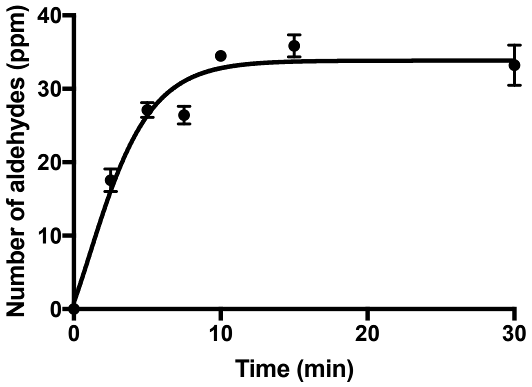

Figure S5. Release of sunscreens from 1:3 AVO/OCR co-encapsulated nanoparticles in 0.25% Tween 20

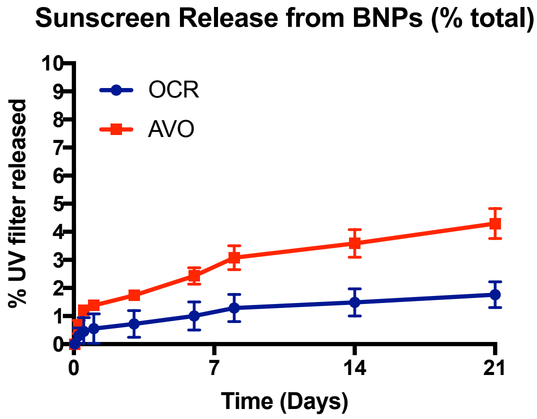

Figure S6. (a) Preparation of Vitro-skin. (b) Fluorescence of PLL treated or untreated VITRO-SKIN® applied with NNPs or BNPs

15% glycerol solution was added to the bottom of a sealable chamber, and VITRO-SKIN® was suspended above the solution to hydrate for 16hrs. For coating with PLL, VITRO-SKIN® was submerged in 0.01% PLL(aq) for 5min, washed with water, and let dry for 15min.

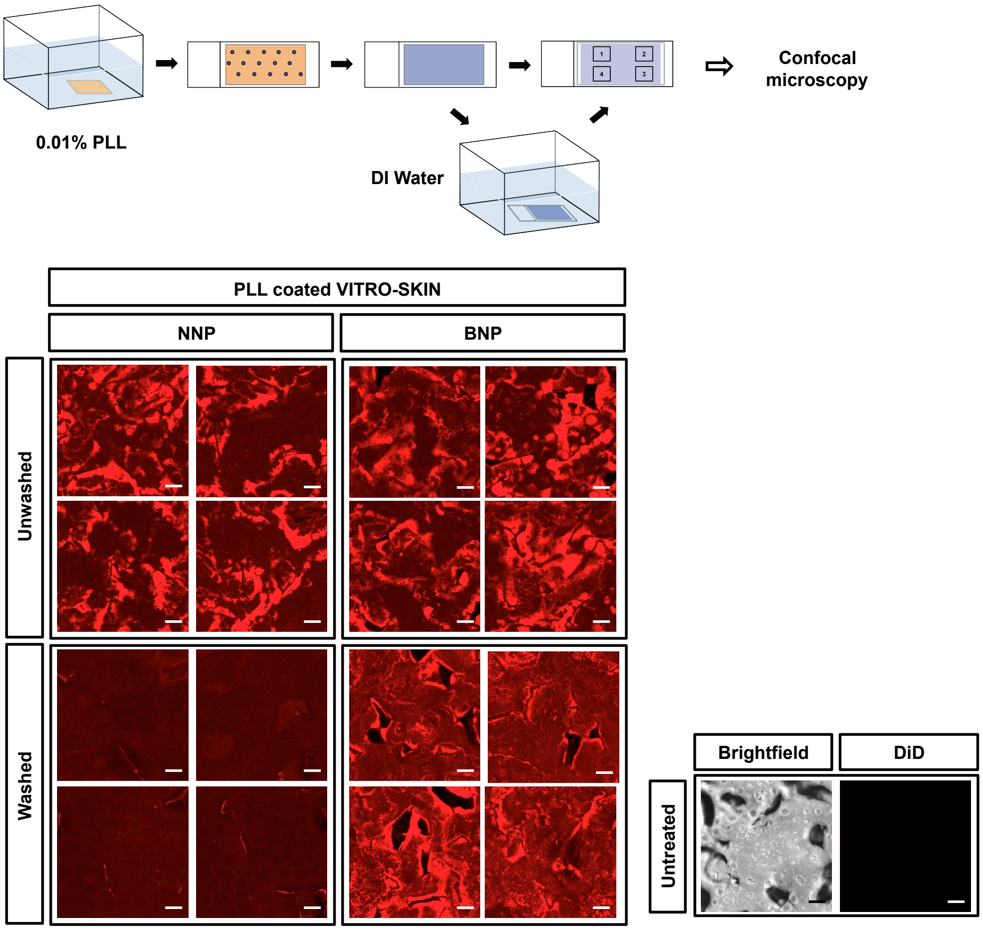

Figure S7. Reproducibility of MEDu

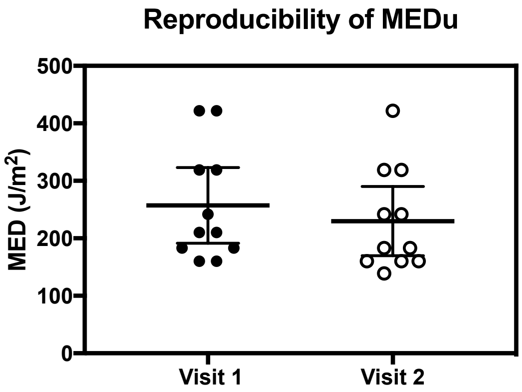

Supplement: Supplementary file 1 — Supporting Information 1 [file BTM2-4-129-s001.pdf]
